# Supplementary material for: Matrix Metalloproteinases in Age-Related Macular Degeneration (AMD)
Source: Int J Mol Sci. 2020 Aug 18;21(16):5934. doi: 10.3390/ijms21165934 (PMC7460693; doi:10.3390/ijms21165934)
Supplement: Supplementary file 1 [file ijms-21-05934-s001.pdf]

Supplementary Table S1. Matrix metalloproteinases classification.

MMPs Classification							
**Traditional classification**	**Numerical classification**	**Chromosomal localization**	**Group of sustrates enzymes**	**General biological effect**	**Reported eye localization**	**Eye implicated processes**	**Ref.**
**Collagenases**							
Collagenase-1	MMP-1	11q22-q23	EMC Substrates<br>Collagen (I, II, III, VII, VIII, X), Entactin, Laminin, Gelatin, Elastin, Fibronectin, Aggrecan, Brevican Neurocan, BM-40, Decorin, Vitronectin, Entactin/ Nidogen, Tenascin, Perlecan, CTGF, Link protein, Myelin basic protein, Fibrin, Fibrinogen	Mediate normal ECM turnover, migration of keratinocytes, re-epithelialization, cell migration, platelet aggregation, increase in the bioavailability of IGF-1, cell proliferation, pro-inflammatory effect, and PARP1 activation	○ Corneal stroma<br>○ Optic nerve head<br>○ Cultured RPE cells<br>○ Trabecular meshwork<br>○ Aqueous humor	AMD Eye Processes<br>- Development of soft drusen- early AMD<br>- The directional shift in the MMP-1/TIMP-1 ratio is associated with increased type I collagen degradation. Potential mechanism contributing to the pathogenesis of exudative AMD	[41,50,54,55]
			Non-EMC Substrates<br>Pro-MMP-1, Pro-MMP-2, proMMP-9, MCP-1, MCP-3, MCP-4, SDF, Pro-1L- 1β, 1L-1β, IL-8, IGFBP-2, IGFBP-3, Pro-TNF-α, CXCL5, CXCL11 precursor, Casein, C1q, Serum amyloid A protein, α_1-Proteinase Inhibitor, α_1-Anti-Chymotrypsin, α_2-Macroglobulin			Non-AMD Eye Processes<br>- Corneal wound repair<br>- Glaucomatous optic nerve head damage<br>- Epiretinal and subretinal membranes in PVR<br>- Activation of fibroblasts at the head of pterygium<br>- Overexpression is related to poor RB differentiation	[49,55–58]
Collagenase-2	MMP-8	11q21-q22	EMC Substrates<br>Collagen (I–III, V, VII, VIII, X), Gelatin, Fibronectin, Laminin Subunit Gamma-	The activation of osteoclasts, the enhancement of collagen affinity, β-	○ Corneal stroma<br>○ Tear samples	AMD Eye Processes<br>- Several roles in ocular inflammation and tissue remolding, including cleavage	

			2, Entactin, Aggrecan, Tenascin, Brevican Core Protein Precursor, Myelin Basic Protein, Fibrinogen	FGF release, anti-inflammatory activity	○ Vitreous samples	degradation ECM components and regulation of cytokine activity	[55,58]
			Non-EMC Substrates			Non-AMD Eye Processes	
			Pro-MMP-8, MCP-1, Pro-TNF-α, IL-8, L-Selectin, IGFBP, CXCL5, CXCL10, CXCL11, Substance P, Angiotensin I, Angiotensin II, Bradykinin, Ephrin- B1, Plasmin C1-Inhibitor, α1-Proteinase Inhibitor, α2-Macroglobulin			- Xerophthalmia<br>- In RD models low MMP-8 expression may lead to inhibition of RPE apoptosis and proliferation<br>- Uveal Melanocytes may participate remolding and inflammatory process via MMP-8	[55,58,59]
			EMC Substrates			Non-AMD Eye Processes	
Collagenase-3	MMP-13	11q22.3	Colagen (I-III, VI, VII, IX, X, XIV), Gelatin, Fibronectin, Laminin Subunit Gamma-2, Collagen telopeptides, SDF, Brevican, Fibrillin, Aggrecan, Perlecan, CTGF, Large Tenascin-C, Osteonectin, SPARC, Biglycan, Fibrinogen	Cell migration, Ability of cleavage intersticial collagens I, II and III, as well as other ECM molecules.<br>Key regulator of choroidal angiogenesis	○ Corneal epithelium	- MMP-13 produced by bone marrow–derived cells appear to be important in experimental choroidal neovascular membrane formation	[55,58,60]
			Non-EMC Substrates			Non-AMD Eye Processes	
			Pro-MMP-9, Pro-MMP-13, MCP-3, SDF, Pro-TNF-α, CXCL5, Factor XII, Casein, C1q, α1-Anti-Chymotrypsin, α2-Macroglobulin			- Corneal wound healing<br>- Increased expression in experimental RD<br>- The modulation of fibrosis could have implications for the development of PVR	[55,56,58,61,62]

						- MMP-13 expressed by pterygium tissues may induce collagen II and III remodeling in pterygium stroma (pterygium recurrence)	
			**Gelatinases**				[32,41,51–53,55,58,63–65]
Gelatinase A	MMP-2	16q13	EMC Substrates<br>Collagen (I, II, III, IV, V, VII, X, XI), Gelatin, Elastin, Fibronectin, Entactin/Nidogen-1, Aggrecan, Decorin, Fibrillin, Fibulin 2, Laminin-5, Tenascin, SPARC, Vitronectin, Galectin-1, Galectin-3, Versican, BM-40, Brevican, Neurocan, CTGF, CSPG-4, Dystroglycan, PCPE-1, Link Protein, Osteonectin, Myelin Basic Protein, Biglycan, Fibrin, Fibrinogen<br>Non-EMC Substrates<br>Pro-MMP-1, Pro-MMP-2, Pro-MMP-13, MMP-12, MCP-3, SDF, Pro-TGF- β1,	The growth of axons, cell migration, the differentiation of mesenchymal cell with inflammation phenotype, enhancement of collagen affinity, cell proliferation, migration of epithelial cells, anti-inflammatory, an increase in the bioavailability of TGF-b, neuronal apoptosis leading to neurodegeneration	○ Cornea (all layers)<br>○ Vitreous<br>○ Optic nerve head<br>○ Interphotoreceptor matrix<br>○ Lens epithelium	AMD Eye Processes<br>- Critical role in the early AMD development, due to the accumulation of deposits under the RPE and increased synthesis of IV type collagen<br>-Total levels of active MMP-2 are significantly reduced in BM from AMD patients<br>- Increase plasma levels in PCV<br>Non-AMD Eye Processes<br>- Corneal wound repair and development<br>- Glaucomatous optic nerve head damage<br>- Vitreous liquefaction	

			Pro-TNF-α, Pro-IL-1β, CXCL5, IGFBP-3, IGFBP-4, IGFBP-5, IGFBP-6, 14-3-3 protein, Big endothelin-1, Cystatin C, Follistatin-like 1, Alpha- actin-2, Pregnancy zone protein, Substance P, Decorin, IGFBP, Plasminogen receptor S100A10, proEMAP/p43, FGF-R1, MMIF, Thrombospondin-2, Plasminogen, α1-Proteinase Inhibitor, α1-Anti-Chymotrypsin, α2-Macroglobulin			- Cataract formation<br>- Uveitis<br>- PDR and new blood vessels formation<br>- MMP-2 inhibition may reduce VEGF expression and, thus, angiogenesis in retinoblastoma cell lines (Overexpression is related to poor RB differentiation)	[55,57,58,63–65]
			EMC Substrates			AMD Eye Processes	
Gelatinase B	MMP-9	20q11.2-q13.1	Collagen (I, IV, V, XI, and XIV), Gelatin, Elastin, Vitronectin, Laminin, Decorin, Fibrillin, Fibronectin, SPARC, Aggrecan, Link Protein, Galectin-1, Galectin-3, Versican, Decorin, Biglycan, Link Protein, Osteonectin, Myelin Basic Protein, Fibrin, Fibrinogen	Collagen affinity enhancing, pro-inflammatory and anti-inflammatory activity, tumor cell resistance, IL-2 response reduction, hypertrophic chondrocyte apoptosis and the subsequent incorporation of new units of functional osteoblasts	○ Corneal epithelium and stroma<br>○ Lens epithelium<br>○ Retinal ganglion cells<br>○ Iris<br>○ Ciliary body<br>○ vitreous	- Increased plasma levels in GA in AMD<br>- Development of CNV as part of AMD pathogenesis<br>- Increase plasma levels in PCV<br>- Total levels of active MMP-9 are significantly reduced in BM from AMD patients	[32,41,51–53,55,58]
			Non-EMC Substrates			Non-AMD Eye Processes	
			Pro-MMP-2, Pro-MMP-9, Pro-MMP-13, MCP-3, SDF, Pro-IL-8, Pro-TNF-α, Pro-TGF-β2, Pro-IL-1β, Cell-surface IL-2Rα, CXCL5,			- Corneal ulcerations and neovascularization<br>- Vitreous liquefaction<br>- Cataract formation<br>- Uveitis	

			CXCL9, CXCL10, CXCL11, FGF-R1, CTAP-III/NAP-2, GROα, PF-4, Integrin beta-2, IGFBP- 1, IGFBP-4, Substance P, Angiotensin I, Angiotensin II, ADAMTS-4, SERPINE2, Casein, C1q, TFPI, Plasminogen, α1-Proteinase Inhibitor, α2-Macroglobulin			- PDR and new blood vessels. An up regulation is associated with CNV. Might be involved in hemorrhagic transformation in patients affected with PDR. Accelerate apoptosis of retinal capillary cells.<br>- MMP-9 inhibition may reduce VEGF expression and, thus, angiogenesis in retinoblastoma cell lines (Overexpression is related to poor RB differentiation)<br>- 17β-estradiol upregulates MMP-9 in the lacrimal gland and conjunctival epithelium, increasing activity in tears of dry subjects<br>- Is linked to and contributes to rod death in RP model	[55,57,58,63–66]
**Stromelysins**							
Stromelysin-1	MMP-3	11q23	EMC Substrates	Migration of cells, epithelial cells apoptosis, the formation of epithelial bubbles, epithelial–mesenchymal conversion, angiostatin-like elements generation, the collagen affinity enhancement, release of bFGF, increase in the bioavailability of IGF-1, cell proliferation, pro-inflammatory and	○ Corneal stroma<br>○ Optic nerve head	AMD Eye Processes	
			Collagen (III, IV, V, VII, IX, X, XI), Gelatin, Collagen Telopeptides, Elastin, Fibronectin, Vitronectin, Laminin, Entactin/ Nidogen-1, Tenascin, SPARC, Aggrecan, Decorin, Perlecan, Versican, Fibulin, Biglycan, Link Protein, Osteonectin, Myelin Basic Protein, Fibulin-2, Fibrin, Fibrinogen			-The directional shift in the MMP-3/TIMP-1 ratio is associated with increased type I collagen degradation. This may be an important mechanism contributing to the pathogenesis of early exudative AMD	[41,55,58]
			Non-EMC Substrates			Non-AMD Eye Processes	
			Pro-MMP-1, Pro-MMP-3, Pro-MMP-7, Pro-MMP-8, Pro-MMP-9, Pro-MMP- 13, MCP-1, MCP-2, MCP-3,			- Autocrine regulation of stromal collagenase in penetrating wounds<br>- Glaucomatous optic nerve head damage<br>- DR	[55,56,63,67]

			MCP-4, SDF, Pro-TNF-α, L-selectin, Pro-HB- EGF, Pro-IL-1β, Perlecan, Decorin, E-cadherin, IGFBP-3, Cm-Tf, Substance P, T-kininogen, Casein, C1q, uPA, uPAR, Osteopontin, VEGFA, PAI, Plasminogen, α2-Antiplasmin, α1-Proteinase Inhibitor, α1-Anti-Chymotrypsin, α2-Macroglobulin	anti-inflammatory activity, increase the bioavailability of TGF-β, disorder in cells aggregation, increase in cell invasiveness; release of VEGF and bFGF, upregulation of angiogenesis		- MMP-3 deficiency reduces leukocyte recruitment to the retina and vitreous cavity<br>-Strong modulator of acute ocular inflammation in the posterior part of the eye<br>- Activation of fibroblasts at the head of pterygium	
Stromelysin-2	MMP-10	11q22.3-q23	EMC Substrates<br>Collagen (I, III, IV, V), Gelatin, Elastin, Fibronectin, Aggrecan, Brevican Hyaluronan and Proteoglycan Link Protein 1, Proteoglycan, Link Protein, Fibrinogen<br>Non-EMC Substrates<br>Pro-MMP-1, Pro-MMP-7, Pro-MMP-8, Pro-MMP-9, Pro-MMP-10, Casein	Generation of tumstatin, endostatin, angiostatin, and endorepellin	○ Corneal epithelium	Non-AMD Eye Processes<br>- Ocular surface diseases<br>- Overexpression in diabetic corneas<br>- Vitrectomy could be associated higher concentrations of MMP-10<br>- PDR	[55,68,69]
Stromelysin-3	MMP-11	22q11.2	EMC Substrates<br>Gelatin, Fibronectin, Collagen IV, Laminin, Aggrecan<br>Non-EMC Substrates<br>Pro-MMP-11, IGFBP-1, Casein, Cm-Tf, α-actin-2, PAI-2, α2-Antiplasmin, α1-	The enzyme is activated intracellularly by furin within the constitutive secretory pathway. Also, in contrast to other MMP's, this enzyme cleaves	○ N/A	- N/A	[55]

			Proteinase Inhibitor, α2-Macroglobulin	alpha 1-proteinase inhibitor but weakly degrades structural proteins of the ECM			
Matrilysin	MMP-7	11q21-q22	EMC Substrates<br>Collagen (IV, V, IX, X, XI), Gelatin, Elastin, Fibronectin, Vitronectin, Laminin, Entactin/ Nidogen-1, Tenascin, SPARC, Aggrecan, Brevican, Galectin-3, Link Protein, Decorin, Fibulin, Versican, Osteonectin, Myelin Basic Protein, Fibrin, Fibrinogen	Adipocyte differentiation, collagen affinity enhancement, an increase in the bioavailability of IGF 1 and TGF-β, cell differentiation, abnormal cell aggregation and increase in cells invasiveness, apoptosis induced by Fas receptor activation, the effect of pro-inflammatory activation of osteoclasts, vasoconstriction and cell growth	○ Corneal epithelium and stroma	AMD Eye Processes<br>- Ability to digest ECM components and cells surface molecules BLD associated with the development of choroidal neovascularization secondary to AMD	[41,55,58]
			Non-EMC Substrates<br>Pro-MMP-1, Pro-MMP-2, Pro-MMP-7, Pro-MMP-9, Pro-TNF-α, Pro-α-defensin, Cell surface bound Fas-L, CXCL9, CXCL11, IGFBP-1, IGFBP-2, IGFBP-3, β4-integrin, E-cadherin, Apo-A1, Apo-CII, CD95-L, Casein, Cm-Tf, Osteopontin, uPA, Plasminogen, Decorin, α1-Proteinase Inhibitor, α2-Macroglobulin			Non-AMD Eye Processes<br>- Corneal wound healing<br>- Human pterygia (angiogenesis)<br>- Increase levels after anti-VEGF in retinal vein occlusion<br>- Elastic fibre degradation in patients with involutional ectropion and entropion	[41,55,70,71]
Metalloelastase	MMP-12	11q22.2-q22.3	EMC Substrates<br>Collagen (I, V, And IV), Gelatin, Elastin,	May be involved in tissue injury and remodeling. Has	○ Corneal epithelium	Non-AMD Eye Processes<br>- Increased expression in RD<br>- Corneal wound healing	[41,55,61,72]

			Fibronectin, Vitronectin, Laminin, Entactin, Osteonectin, Decorin, Aggrecan, Biglycan, Fibrillin, Myelin Basic Protein, Fibrin, Fibrinogen<br>Non-EMC Substrates<br>Plasminogen, Pro-TNF-α, ApoA-1, CXCL9, CXCL10, CXCL11, uPAR, vWF, Factor XII, TFPI, α1-Proteinase Inhibitor	significant elastolytic activity		- May be involved in the ECM remodeling<br>- Kew regulator of macrophage infiltration and inflammation, contributing to retinal vascular dysfunction and pathological angiogenesis. The increase or decrease of its levels facilities CNV formation<br>- The modulation of fibrosis could have implications for the development of PVR	
Matrilysin-2	MMP-26	11p15	EMC Substrates<br>Collagen IV, Gelatin, Fibronectin, Vitronectin, Fibrinogen<br>Non-EMC Substrates<br>Pro-MMP-9, Pro-MMP26, IGFBP-1, α1-Proteinase Inhibitor, α2-Macroglobulin	Ability to digest ECM components	○ epithelial conjunctiva cells	Non-AMD Eye Processes<br>- Expression in glioma is correlated with poor clinical outcome	[55]
Enamelysin	MMP-20	11q22.3	EMC Substrates<br>Collagen XVIII, Amelogenin, Ameloblastin, Aggrecan, Laminin, COMP<br>Non-EMC Substrates<br>Pro-MMP-20 (autolysis)	May play a central role in tooth enamel formation	○ N/A	AMD Eye Processes<br>- Not associated with the appearance of exudative AMD, but it could affect the size of the neovascular lesion	[38]
**Membrane-type MMPs (MT-MMP)**							
MT-MMP-1	MMP-14	14q11-q12	EMC Substrates<br>Collagen (I, II and III), Gelatin, Fibronectin, Tenascin, Vitronectin, Laminin, Entactin,	Anti-inflammatory, cell migration, the formation of renal tubules, epithelial cell migration,	○ Corneal epithelium<br>○ Human TM cell culture,	Non-AMD Eye Processes<br>- Corneal infection, wound healing, inducing corneal neovascularization (pro-angiogenic factor)<br>- Proliferative diabetic retinopathy	[41,49,55,61,72]

			Galectin-3, CTGF-L, Fibrillin, Aggrecan, Perlecan, Syndecan-1, Lumican, Myelin Basic Protein, Fibrinogen<br>Non-EMC Substrates<br>Pro-MMP-2, Pro-MMP-13, Pro-MMP-14, MMP-14, MCP-3, SDF, Cell-surface CD44, RANKL, tTG, Pro-TNF-α, IL-8, HB-EGF, Factor XII, Pro-αv integrin chain, Apo-CII, Apo-E, α1-Proteinase Inhibitor, α2-Macroglobulin	adhesion reduction, flattening of the cells reduction, trailers embryo to the uterine epithelium<br>- Activation MMP-2	human TM explant culture	- Implications for ECM turnover<br>- Biomarker of angiogenic activity in PDR<br>- Keratinocyte migration	
MT-MMP-2	MMP-15	15q13-q21	EMC Substrates<br>Fibronectin, Tenascin, Entactin, Laminin, Aggrecan, Perlecan, Proteoglycan, Myelin Basic Protein<br>Non-EMC Substrates<br>Pro-MMP-2, Cell-surface bound tTG, Pro-TNF-α	Adhesion and cell flattering reduction	○ Human TM cell culture, human TM explant culture	Non-AMD Eye Processes<br>-Could play a modulatory role in IOP homeostasis	[55,73,74]
MT-MMP-3	MMP-16	8q21	EMC Substrates<br>Collagen III, Gelatin, Fibronectin, Vitronectin, Laminin, Myelin Basic Protein<br>Non-EMC Substrates<br>Pro-MMP-2, Cell-surface bound tTG, Pro-TNF-α,	Adhesion and cell flattering reduction	○ Human TM cell culture, human TM explant culture	Non-AMD Eye Processes<br>-Could play a modulatory role in IOP homeostasis	[55,73,74]

			Metastasis-suppressor KiSS-1				
MT-MMP-4	MMP-17	12q24.3	EMC Substrates<br>Gelatin, Fibrin, Fibrinogen, Myelin Basic Protein<br>Non-EMC Substrates<br>Pro-MMP-2, Pro-TNF-α, HB-EGF, ADAMTS4 Peptidase	May be involved in the activation of membrane-bound precursors of growth factors or inflammatory mediators, such as TNF-α. May also be involved in tumoral process.	○ Human TM cell culture, human TM explant culture	Non-AMD Eye Processes<br>-Could play a modulatory role in IOP homeostasis	[55,73,74]
MT-MMP-5	MMP-24	20q11.2	EMC Substrates<br>Fibronectin, Gelatin, Chondroitin Sulphate Proteoglycan, Dermatan Sulphate Proteoglycan<br>Non-EMC Substrates<br>Pro-MMP-2, KiSS-1	Involved in cell-cell interactions between nociceptive neurites and mast cells. May play a role in axonal growth	○ Human TM cell culture, human TM explant culture	Non-AMD Eye Processes<br>-Could play a modulatory role in IOP homeostasis	[55,73,74]
**Tissue inhibitor of metalloproteinases (TIMPs)**							
Metalloproteinase inhibitor 1	TIMP-1	Xp11.3	All types of MMPs (MMP-1, MMP-3, MMP-7 and MMP-9) and MT-MMPs	Connective tissue cells, macrophages Increased expression has been found associated with worse prognosis of tumors such as laryngeal carcinoma or melanoma	○ Corneal epithelium and endothelium<br>○ Optic nerve head<br>○ Retinal ganglion cells<br>○ Vitreous<br>○ IPM	AMD Eye Processes<br>- Potential role for MMPs in the development of CNV in AMD. TIMP-1 promotes VEGF-induced neovascularization in the retina<br>- Increase levels in patients with GA in AMD<br>- Lower TIMP-3 levels are associated with higher TIMP-1 levels and the presence of GA in AMD	[16,17,51,52]
						Non-AMD Eye Processes<br>-Increased expression in experimental RD and PVR	[61,72,75,76]

						- Corneal wound healing, keratoconus<br>- POAG	
Metalloproteinase inhibitor 2	TIMP-2	17q25.3	All types of MMPs (MMP-2)	Connective tissue cells, macrophages Important role in hippocampus and cognitive function It has an independent antiangiogenic effect	○ Corneal epithelium<br>○ RPE<br>○ Retinal ganglion cells<br>○ IPM<br>○ Vitreous	AMD Eye Processes<br>- In combination with MMP-14, is involved in regulation of MMP-2. High levels of TIMP-2 inhibit MMP-2 activity- Potential role in AMD	[16,17,51,52]
						Non-AMD Eye Processes<br>- Potential Biomarkers of RB progression<br>- Increased expression in experimental RD and PVR<br>- Corneal wound healing, keratoconus<br>- POAG	[57,61,72]
Metalloproteinase inhibitor 3	TIMP-3	22q12.3	All types of MMPs<br>ADAM<br>ADAMTS	Blocks the development of neovascularization by inhibiting the binding of VEGF to the VEGF receptor, Regulate local MMP to maintain rate of turnover and limit choroidal growth Fibroblasts, synovial cells	○ RPE<br>○ BM<br>○ Corneal epithelium<br>○ Vitreous<br>○ IPM	AMD Eye Processes<br>- Excess TIMP-3, may retard BM renewal and result in the thickening.<br>- Reduced TIMP-3 production, causes an increase in the amounts of various collagens and development of early dry AMD.<br>Also increases ADAM/ADAMTs, which can be implicated in CNV formation in AMD<br>-Mutations in this gene have been associated with the autosomal dominant disorder SFD	[16,17,51,52]
						Non-AMD Eye Processes<br>- Potential role in RP	[17,77]
Metalloproteinase inhibitor 4	TIMP-4	3p25.2	MMP-1, MMP-2, MMP-3, MMP-7 and MMP-9	Role in the regulation of platelet aggregation, hormonal and	○ Aqueous humor	Non-AMD Eye Processes<br>- POAG	[76]

endometrial tissue remodeling

Ref: references. MMP-1: matrix metalloproteinase 1; BM-40: basement-membrane protein 40; CTGF: CCN2 or connective tissue growth factor ; ECM: extracellular matrix ; IGF-1: insulin-like growth factor 1; MCP-1: Monocyte chemoattractant protein-1; SDF: stromal cell derived factor ; AMD: age-related macular degeneration; RPE: retinal pigment epithelium; IGFBP: insulin like growth factor binding protein; CXCL11 precursor: C-X-C motif chemokine 11; PARP1: Poly [ADP-ribose] polymerase 1; TNF-α : Tumor necrosis factor alpha; TIMP-1: metalloproteinase tissue inhibitor 1 ; RB: retinoblastoma; MMP-8: matrix metalloproteinase 8; β-FGF: basic fibroblast growth factor; RD: retinal detachment; MMP-13: matrix metalloproteinase 13; SPARC: Secreted protein acidic and rich in cysteine; PVR: proliferative vitreoretinopathy; MMP-2: matrix metalloproteinase 2; CSPG-4: chondroitin sulfate proteoglycan 4; PCPE-1: procollagen c-proteinase enhancer 1; PCV: polypoidal choroidal vasculopathy; PDR: proliferative diabetic retinopathy ; VEGF: vascular endothelial growth factor; proEMAP/p43: tumour-derived cytokine; FGF-R1: fibroblast growth factor receptor 1; MMIF: macrophage migration inhibitory factor; TGF-b: transforming growth factor beta; MMP-9: matrix metalloproteinase 9; IL: interleukin; FGF-R1: fibroblast growth factor receptor 1; CTAP-III/NAP-2: connective tissue-activating peptide III/neutrophil-activating peptide-2; GROα: growth-regulated oncogene alpha; PF-4: platelet factor 4; ADAMTS-4: a disintegrin and metalloproteinase with thrombospondin motifs; SERPINE2: serpin family E member 2; TFPI: tissue factor pathway inhibitor; CNV: choroidal neovascularization; GA: geographic atrophy; RP: retinitis pigmentosa; MMP-3:matrix metalloproteinase 3; Pro-HB- EGF: heparin-binding epidermal growth factor-like growth factor; Cm-Tf: carboxymethylated transferrin; uPA: urokinase plasminogen activator; uPAR: urokinase plasminogen activator receptor ; MMP-10: matrix metalloproteinase 10; MMP-11: matrix metalloproteinase 11; N/A: not available; PAI-2: plasminogen activator inhibitor-2; MMP-7: matrix metalloproteinase 7; BLD: basal laminar and linear deposits; Fas-L: Fas ligand; Apo-A1: apolipoprotein A1; Apo-CII: apolipoprotein C-2;CD95-L: cluster of differentiation-95 ligand; MMP-12: matrix metalloproteinase 12; vWF: von Willebrand factor; MMP-26: matrix metalloproteinase 26; MMP-20: matrix metalloproteinase 20; COMP: cartilage oligomeric matrix protein; MMP-14: matrix metalloproteinase 14; CTGF-L: connective tissue growth factor-like; RANKL: receptor activator of nuclear factor kappa-B ligand; tTG: tissue transglutaminase; TM: trabecular meshwork; MMP-15: matrix metalloproteinase 15; IOP: intraocular pressure; MMP-16: matrix metalloproteinase 16; Metastasis-suppressor KiSS-1: metastasis-suppressor kisspeptin 1; MMP-17: matrix metalloproteinase 17; MMP-24: matrix metalloproteinase 24; IPM: interphotoreceptor matrix; ADAM: a disintegrin and metalloproteinase; ADAMTS: A Disintegrin-like And Metalloproteinase with ThromboSpondin motifs; TIMP-2: metalloproteinase tissue inhibitor 2; TIMP-3: metalloproteinase tissue inhibitor 3; TIMP-4: metalloproteinase tissue inhibitor 4; SFD: Sorsby's Fundus Dystrophy; POAG: primary open angle glaucoma.
